# Human Placental Mesenchymal Stem/Stromal cells (pMSCs) inhibit agonist‐induced platelet functions reducing atherosclerosis and thrombosis phenotypes

**DOI:** 10.1111/jcmm.16848

**Published:** 2021-09-18

**Authors:** Abdullah Al Subayyil, Yasser S. Basmaeil, Reem Alenzi, Tanvir Khatlani

**Affiliations:** ^1^ Stem Cells and Regenerative Medicine Department King Abdullah International Medical Research Center King Saud bin Abdulaziz University for Health Sciences, King Abdulaziz Medical City, Ministry of National Guard Health Affairs Riyadh Saudi Arabia

**Keywords:** atherosclerosis, CD36, flow cytometry, ox‐LDL, placental Mesenchymal Stem/Stromal cells, platelet activation, platelet aggregation, platelet spreading

## Abstract

Mesenchymal stem/stromal cells isolated from human term placenta (pMSCs) have potential to treat clinically manifested inflammatory diseases. Atherosclerosis is a chronic inflammatory disease, and platelets play a contributory role towards its pathogenesis. During transplantation, MSCs interact with platelets and exert influence on their functional outcome. In this study, we investigated the consequences of interaction between pMSCs and platelets, and its impact on platelet‐mediated atherosclerosis in vitro. Human platelets were treated with various types of pMSCs either directly or with their secretome, and their effect on agonist‐mediated platelet activation and functional characteristics were evaluated. Human umbilical vein endothelial cells (HUVECs) were used as control. The impact of pMSCs treatment on platelets was evaluated by the expression of activation markers and by platelet functional analysis. A subset of pMSCs reduced agonist‐induced activation of platelets, both via direct contact and with secretome treatments. Decrease in platelet activation translated into diminished spreading, limited adhesion and minimized aggregation. In addition, pMSCs decreased oxidized LDL (ox‐LDL)‐inducedCD36‐mediated platelet activation, establishing their protective role in atherosclerosis. Gene expression and protein analysis show that pMSCs express pro‐ and anti‐thrombotic proteins, which might be responsible for the modulation of agonist‐induced platelet functions. These data suggest the therapeutic benefits of pMSCs in atherosclerosis.

## 1 INTRODUCTION

Cardiovascular diseases (CVDs) are the number one cause of global death and disability. According to WHO, an estimated 17.9 million lives are consumed each year by the CVDs.^1^ CVDs represent a group of disorders involving heart and blood vessels. It is mainly associated with atherosclerosis and an increased risk of blood clots.^1^ Atherosclerosis is typically characterized by plaque formation affecting different vascular beds.^2^, ^3^, ^4^ Platelet activation and hyper‐reactivity have been thought to contribute towards pathogenesis of acute atherothrombotic disorders, such as myocardial infarction (MI) and stroke.^5^ Although the classical role of platelets is maintenance of haemostasis, yet from recent investigations, it has become evident that they play a central role in promoting inflammation through their interactions with leukocytes.^6^, ^7^, ^8^ These interactions make them relevant in the pathophysiology of atherosclerosis, which is now considered an inflammatory disorder.^7^, ^9^, ^10^, ^11^ Clinical studies also support an association between platelet reactivity and prognosis in patients with coronary disease.^12^ In addition, numerous studies have linked platelets and hyperlipidaemia to atherothrombotic risk.^13^ Drugs causing specific inhibition of platelet function are important for treatment of cardiovascular and cerebrovascular diseases.^2^

Platelets express multiple receptors on its surface, which facilitate relevant physiological processes during haemostasis.^14^, ^15^, ^16^ At the site of vascular injury, the endothelial lining and the damaged tissue generate adhesion molecules and soluble platelet agonists, such as collagen, von Willebrand factor (vWF), adenosine diphosphate (ADP) and thrombin. They interact with specific receptors on the platelet surface and initiate platelet activation followed by adhesion, activation and aggregation, thereby promoting plug formation and thrombus development.^17^, ^18^ CD36 (also called as glycoprotein IV) is a multi‐ligand receptor expressed in macrophages, dendritic cells (DCs) and in platelets, which recognizes certain oxidized phospholipids.^5^ Recent studies have indicated that CD36 binding to oxidized LDL (ox‐LDL) results in activation of specific signalling cascade that induces platelet activation.^19^, ^20^ This binding and activation is believed to contribute to the pro‐coagulant state associated with hyperlipidaemia and contribute to atherosclerosis.^21^, ^22^

Mesenchymal stem/stromal cells (MSCs) have emerged as an option in cell‐based therapy for many diseases, including diabetes, cancer, CVDs and atherosclerosis.^23^, ^24^, ^25^, ^26^ MSCs are multipotent adult stem cells isolated from different tissues including adipose, dental pulp, bone marrow, umbilical cord and placenta. Their therapeutic potential has been attributed to their differentiation potential, immunomodulatory capabilities and tissue regeneration properties.^27^ We have isolated and characterized MSCs from various parts of human term placenta (pMSCs) including, *decidua basalis* (DBMSCs), *decidua parietals* (DPMSCs) and *chorionic villous* (CVMSCs) regions.^28^, ^29^, ^30^ pMSCs exhibit immunomodulatory properties, making them excellent sources for cell‐based therapies against immune‐mediated diseases. By secreting soluble factors, they exert immune responses on T cells, B cells, monocytes and macrophages.^31^ We have previously reported the therapeutic potential of pMSCs in treating inflammatory diseases such as cancer and diabetes,^32^, ^33^ and in this study, we investigated if pMSCs modulate platelet functions and in turn play any restrictive role towards pathogenesis of atherosclerosis and thrombosis. Towards that goal, we studied the effect of pMSCs on platelet activation in presence and absence of various agonists. The effect of pMSCs at both intracellular contact (IC) and their secretome levels (using conditioned medium (CM)) was evaluated and compared their effect to those of human umbilical vascular endothelial cells (HUVECs). In addition, we studied the effect of pMSCs on ox‐LDL‐induced‐CD36‐mediated aggregation of platelets, using the standard platelet‐specific functional assays. DBMSCs and DPMSCs but not CVMSCs reduced agonist‐induced activation of platelets significantly, both via the IC and with the CM treatments. However, no change was observed in the resting platelets. Decrease in platelet activation translated into their diminished spreading, limited adhesion and minimized aggregation. Most strikingly, pMSCs decreased ox‐LDL‐induced‐CD36‐mediated platelet activation significantly, as compared to resting and/or pMSCs only treated platelets. pMSCs express pro‐ and anti‐thrombotic proteins, which may be responsible for such outcome. However, their specific roles and mechanism of action need further investigation.

## 2 MATERIAL AND METHODS

### 2.1 Experimental ethics

This study was approved by the Institutional Review Board (IRB) of King Abdullah International Medical Research Center (KAIMRC), Riyadh, KSA. Voluntary healthy donors were asked to sign an informed consent as approved by the IRB, before collection of the samples.

### 2.2 Reagents and antibodies

Agonists ADP, and collagen (cat#5852), were purchased from Helena Laboratories Corporation. U46619 (cat#538944‐Calbiochem) was purchased from Sigma‐Aldrich. Alexa Fluor‐488‐conjugated fibrinogen (catalog# F13191) was purchased from Molecular Probes, Inc. Thermo Fisher Scientific. Human Fibrinogen (cat#16088) was purchased from Cayman Chemical Company. Fluorescein isothiocyanate (FITC)‐conjugated Mouse Anti‐Human CD62P, Clone AK‐4 (cat# 555523) also called as P‐selectin and FITC Mouse Anti‐Human PAC1 (cat# 340507) specifically binding to human activated α_IIb_β_3_ (CD41/CD61) complex were purchased from BD Biosciences. Control FITC tagged IgG, FITC Mouse Anti‐Human IgG Clone G18‐145 (cat#555786) was purchased from BD Bioscience. Primary antibodies to Human GP VI (cat# AF3627); Human/Mouse PLC‐gamma 2 (cat# MAB3716); Human TFPAI (cat#AF2974) and Human Protein S/PROS1 (cat# MAB4036) were purchased from R&D Systems, whereas antibodies to PROC1 (catalog# 25382‐1‐AP) and Low Density Lipoprotein from Human Plasma, oxidized (OxLDL) kit (cat# L34357) were purchased from Thermo Fisher Scientific. Goat anti‐rabbit and goat anti‐mouse secondary antibodies were purchased from Sigma‐Aldrich, whereas β‐Actin antibody was purchased from Cell Signaling Technology.

### 2.3 Collection and processing of human placentae and umbilical cord tissues

Human placentae and umbilical cord tissues were collected from healthy donors with uncomplicated gestation and with normal vaginal delivery after 38–40 weeks of gestation. Foetal viability and age of gestation were confirmed by ultrasound examinations during the gestation period. The tissues were processed within 2 h of delivery following proper experimental procedures and guidelines.

### 2.4 Blood collection and platelet isolation

Human blood was collected from healthy and fasting volunteers in anticoagulant acid citrate dextrose (ACD) containing tubes. Platelets were isolated as described earlier.^34^ Briefly, the tubes were centrifuged at 900 RPM for 15 min (min) at room temperature and platelet‐rich plasma (PRP) was collected. Prostaglandin E_1_ (PGE_1_) at 75 nM was added to the PRP before centrifuging again for 10 min at 420*g*. Supernatant was discarded, and the pellet was resuspended in citrate glucose sodium chloride (CGS) solution containing PGE_1_ at 75 nM final concentration. The suspension was centrifuged at 420*g* for 10 min, supernatant was discarded, and the platelets were resuspended in Tyrode's Buffer (138 mm NaCl, 5.3 mm KCl, 0.33 mm Na_2_HPO_4_, 0.44 mm KH_2_PO_4_, 5.5 mm glucose, pH 7.4) and centrifuged. The supernatant was discarded, and washed platelet pellets were suspended in Tyrode's buffer without PGE_1_, calcium and magnesium, and adjusted to a final concentration of 2.5 × 10^8^ platelets/ml. Washed platelets were rested at room temperature for 30 min before use.

### 2.5 Platelet treatment and flow cytometry

Washed platelets diluted to 2.5 × 10^8^/ml in Tyrode's buffer containing 1.8 mM CaCl_2_ and 0.49 mM MgCl_2_ were incubated with respective MSCs and HUVECs (corresponding to 1:1; 1:2 and 1:5 ratio between platelets and pMSCs) or CM (at 5%, 10% or 25% final concentration) for 10 min at room temperature. The incubated platelets were activated with collagen (1 μg/ml and 2.5 μg/ml), ADP (1 μM and 5 μM) or U46619 (5 μM), for 10 min at room temperature, before subjecting to flow cytometry for evaluation of activation markers and other functional analyses.

For assessment of surface antigens and other activation markers, the MSCs‐treated, MSCs‐untreated and resting platelets were incubated with Alexa 488‐conjugated fibrinogen, FITC‐conjugated CD62P, PE‐conjugated CD41, FITC Mouse Anti‐Human PAC1, FITC or PE tagged isotype control antibodies and assayed by BD FACS CANTO II flow cytometer (Becton Dickinson). The results were analysed with FlowJo software (FlowJo), as described earlier.^35^

### 2.6 Platelet spreading, adhesion and light transmission aggregometry

Resting and pMSCs/HUVEC and CM‐treated platelets (agonist treated or untreated) at 1 × 10^5^/ml were incubated on human fibrinogen (Enzyme Research)‐coated glass coverslips (Corning), for 15 and 30 min at 37°C. Excessive protein and non‐adhered platelets were washed out by rinsing. Adhered platelets were fixed with 3.7% paraformaldehyde and permeabilized with 0.01% Triton X‐100. Fixed platelets were stained for F‐actin by Rhodamine Phalloidin. The slides were visualized and photographed using a bright field microscope. We have visualized 10 slides, and 5 to 10 pictures were captured from each slide for further analysis. The surface area of the platelets was quantified using the ImageJ software^®^ (NIH, USA).

For adhesion assay, pMSCs/HUVEC and CM‐treated and CM‐untreated platelets were challenged with appropriate agonists before adding to the fibrinogen‐coated 96‐well plates. The wells were blocked with BSA and incubated at 37°C for 30 and 60 min. After washing the wells, platelet adhesion was quantified by acid phosphatase activity assay, and OD was measured at 405 nM, as described earlier.^36^

Aggregation assays for pMSCs/HUVEC and CM‐treated and CM‐untreated platelets followed by challenging with different doses of agonists (collagen and ADP) were evaluated in stirring conditions in an eight channel aggregometer, PAP‐8E (Bio/Data Corporation). The samples were heated to 37°C for 5 min before subjecting to analysis. Aggregation was recorded following the degree of electrical impedance in washed and treated samples. Samples were run for 10 min, and data were recorded when the level of aggregation had reached the maximum extent.

*For methods of isolation and culture of pMSCs and HUVECs*, *collection of conditioned media (CM)*, *real*‐*time PCR (RT*‐*PCR)*, *immunoblotting and statistical analysis*, *please refer to the* Supplementary Methods.

## 3 RESULTS

### 3.1 Dose response for CM treatment on platelet activation

**FIGURE 1:**
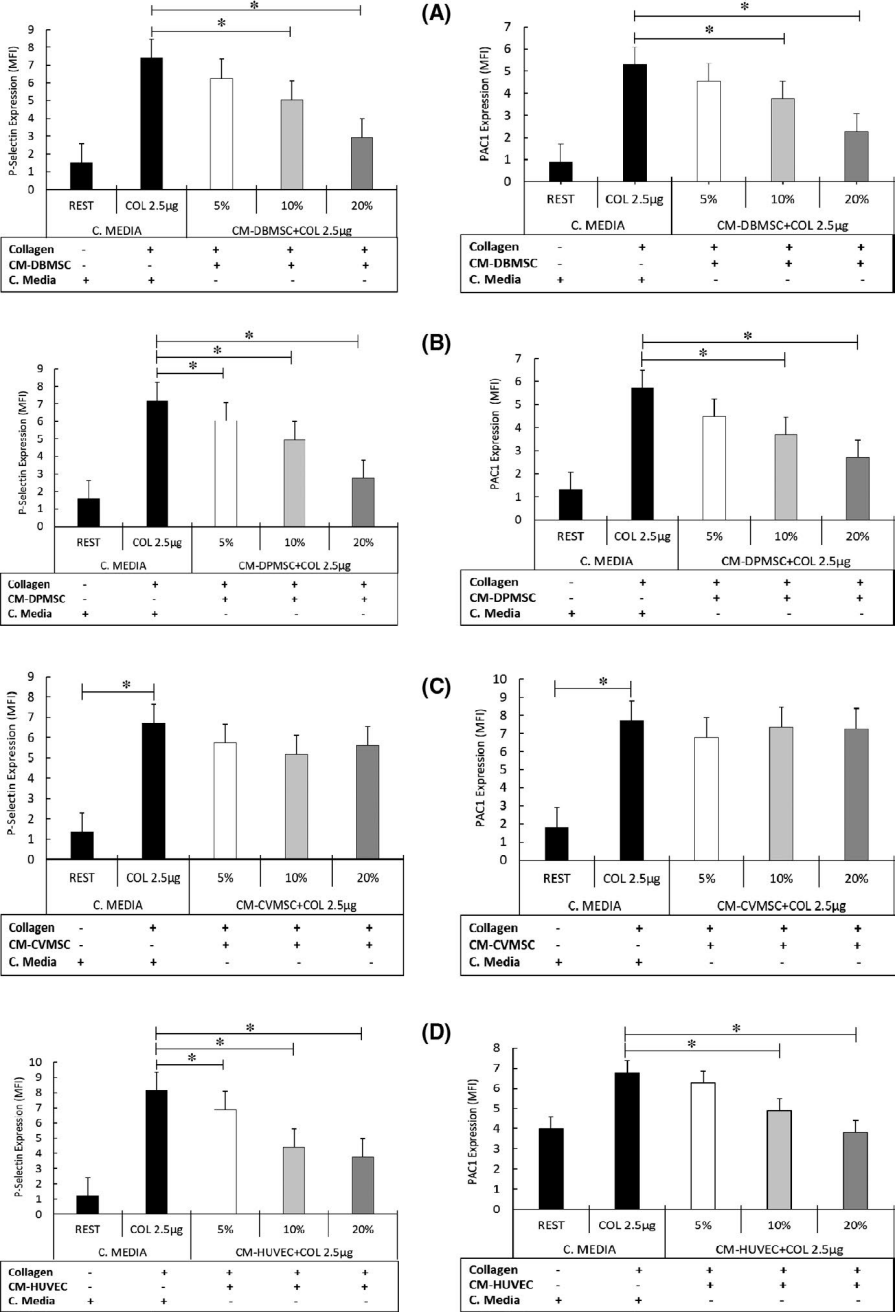
Conditioned media of pMSCs inhibit platelet activation: Washed platelets incubated with conditioned medium (CM) at 5%, 10% and 20% concentration and then induced with collagen. Flow cytometry was performed to assess the expression of activation markers, P‐selectin (CD62P) and PAC1. (A) CM‐DBMSCs; (B) CM‐DPMSCs; (C) CM‐CVMSCs; (D) CM‐HUVECs. Data are expressed as Mean Fluorescent Index (MFI). Platelet MFI after treatment with different CM concentrations and agonists is compared with CM and collagen untreated and washed platelets (Rest) as negative and collagen‐treated platelets as positive control. Bars with standard error (±SE) are representative of at least three experiments with pMSCs isolated from at least five different placentae. *p ≤ 0.05

Agonists (Collagen, U46619 and ADP) were added to the pMSCs CM‐treated platelets at different doses (collagen (1 μg/ml and 2.5 μg/ml), ADP (1 μM and 5 μM) and U46619 at 10 μM) and incubated for staggered time points ranging from 2–10 min. As shown in Figure 1, the expression of activation marker P‐selectin and PAC1 decreased robustly in a dose‐dependent manner, ranging from 5% to 20% as compared to the collagen‐treated (CM‐untreated) control. This change in expression levels was significant (*p *< 0.05) for 10% and 20% compared to the 5% treatment in CM‐DBMSCs (Figure 1A) as well as for CM‐DPMSCs (Figure 1B)‐treated platelets. However, for CM‐CVMSCs (Figure 1C), P‐selectin expression showed decreased levels, yet it was not statistically significant, as compared to collagen‐treated (CM‐untreated) control. There was no change in the PAC1 expression levels in platelets treated with CM‐CVMSCs and collagen. As expected, the expression of P‐selectin decreased significantly (*p *< 0.05) for all tested doses, whereas PAC1 expression in platelets decreased significantly (*p *< 0.05), for 10% and 20% HUVECs CM (Figure 1D), compared to the 5% treatment conditions.

Contrary to the results obtained for collagen‐induced platelet activation, ADP‐induced and pMSCs CM‐treated platelets exhibited slightly different expression profile for P‐selectin and PAC1. As shown in supplementary figure S1, although CM of DBMSCs exhibited reduced P‐selectin and PAC1 expression in a dose‐dependent manner (Figure S1A) yet the results were not statistically significant, as compared to ADP‐treated (CM‐untreated) control. Platelets treated with CM‐DPMSCs and CM‐CVMSCs, and activated with ADP did not show any change in the expression levels of the P‐selectin or the PAC1 (Figure S1B and C). However, as expected, HUVECs CM‐treated platelets and activated with ADP showed significant reduction (*p *< 0.05) in the expression of P‐selectin as well as PAC1, in a dose‐dependent manner (Figure S1D).

In resting (CM and agonist untreated) platelets, the expression levels of both P‐selectin and PAC1 were significantly low (*p *< 0.05) as compared to the agonist treated and activated platelets.

### 3.2 pMSCs partially inhibit agonist‐induced platelet activation

**FIGURE 2:**
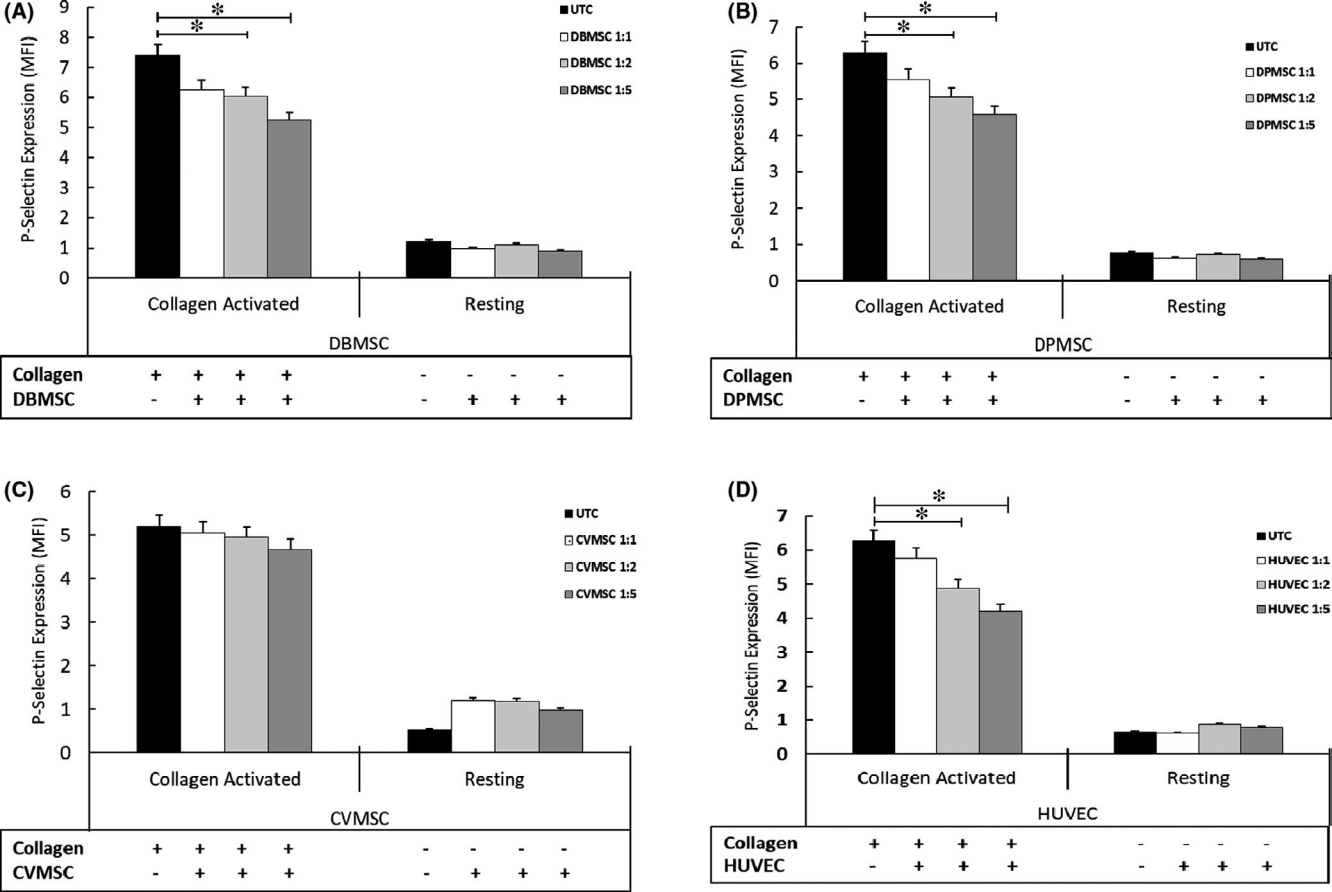
Dose response for pMSCs treatment on platelet activation: Platelets were co‐cultured (intracellular treatment IC) with varied number of pMSCs corresponding to 1:1, 1:2 and 1:5 ratio between platelets and stem cells, and subsequently induced with collagen at 2.5 µM or left as untreated controls. The activation of platelets was assessed by flow cytometry for activation marker P‐selectin. Dose effect of pMSCs treatment on platelet activation as compared to pMSCs‐untreated and collagen‐treated and resting platelets. (A) DBMSCs; (B) DPMSCs; (C) and (D) CVMSCs and HUVECs, respectively. Data are reflected as MFI. Data are represented as bars with ±SE. Figure is representative of three individual experiments. pMSCs isolated from 5 different placentae for each cell type were used in the study. *p ≤ 0.05

DBMSCs and DPMSCs inhibited activation of collagen‐treated platelets at all cellular ratios tested in the co‐culture experiments. As shown in Figure 2A and B, there was a significant (*p *< 0.05) decrease in activation at the 1:2 and 1:5 (platelet to MSCs) ratio, as compared to untreated control. However, as observed for CM‐CVMSCs, the cellular component of CVMSCs also did not inhibit the collagen‐induced activation of platelets (Figure 2C). Similarly, as CM of HUVECs had inhibited the collagen‐induced platelet activation, HUVECs also decreased the collagen‐induced activation of platelets in a dose‐dependent manner (Figure 2D), as compared to untreated control.

Similar experiments were performed on resting platelets to examine whether pMSCs and HUVECs modulate activation of the resting platelets. Figure 2 (A, B and D) shows that DBMSCs, DPMSCs and HUVECs did not activate the resting platelets. Interestingly, slight increase (~2 fold) in P‐selectin expression was observed in platelets treated with CVMSCs as compared to the untreated control. However, the expression levels were not statistically significant as compared to the collagen‐induced and MSC‐treated or MSC‐untreated platelets (Figure 2C). As shown in the supplementary figure S2A, although DBMSCs reduced the ADP‐induced activation of platelets at all cellular ratios used in the study, yet the data were not statistically significant as compared to untreated control. DPMSCs and the CVMSCs did not alter the ADP‐induced activation for any cellular ratios or platelet numbers tested in the experiments (Figure S2B and C). The resting platelets treated with both pMSCs and HUVECs did not show any increase in the P‐selectin levels at any ratio of cells used in the study.

**FIGURE 3:**
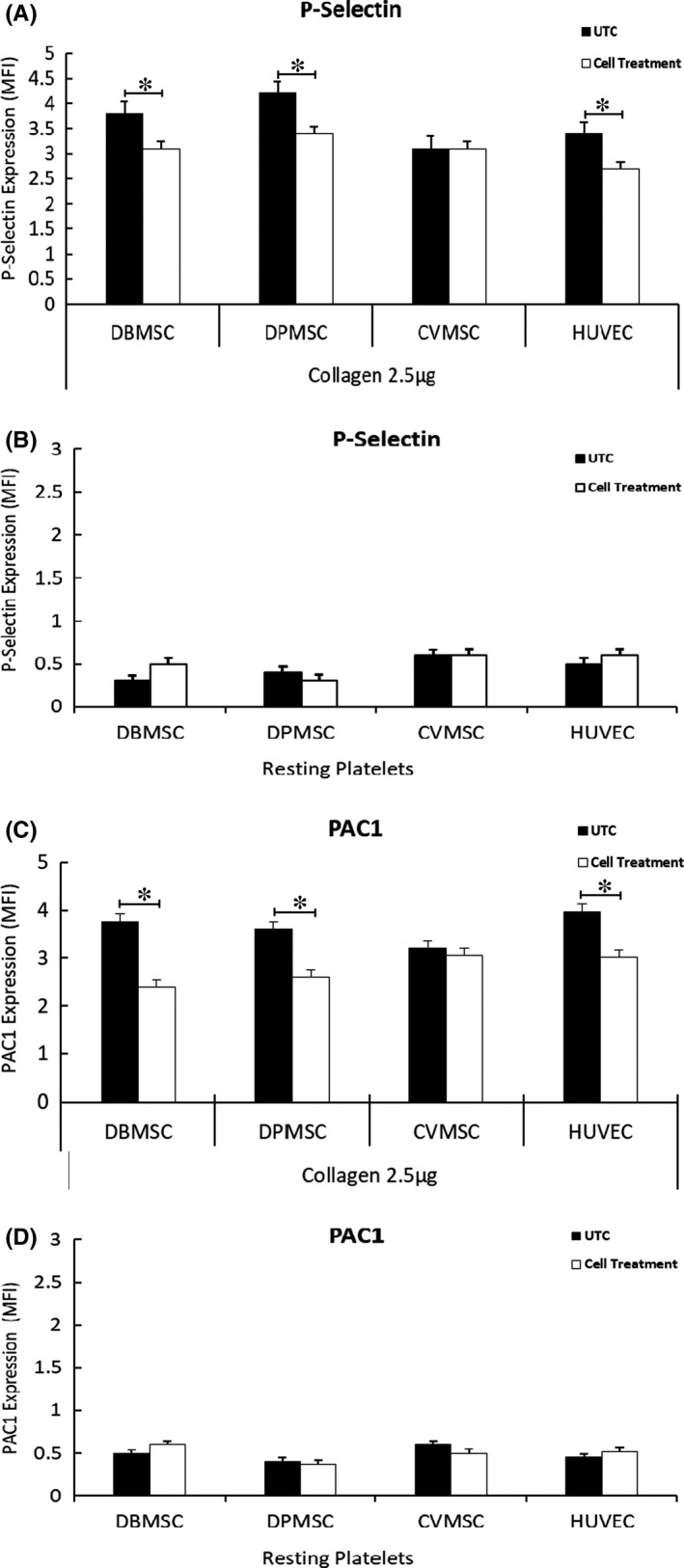
A subset of pMSCs inhibit platelet activation: Platelets co‐cultured with or without (w/o) pMSCs/HUVECs followed by activation with collagen or the resting (untreated) followed by flow cytometry for activation markers P‐selectin (CD62P) and PAC1. P‐selectin (CD62P) expression in MFI for: (A) pMSCs/HUVECs‐treated and collagen‐induced, (B) pMSCs/HUVECs‐treated and resting platelets. PAC1 expression in MFI for: (C) pMSCs/HUVECs‐treated, collagen‐induced, (D) pMSCs‐treated, and resting platelets. Data are represented as bars with SE. Figure is representative of three separate experiments performed with pMSCs or HUVECs isolated from 5 different placentae for each cell type used in the study. *p ≤ 0.05. UTC, untreated cellular control

As 2.5 × 10^6^ cells are an agreed dose necessary for successful transplantation in clinical settings,^37^ we used these cell numbers against specific number of platelets to achieve a ratio of 1:5. As shown in Figure 3, the expression levels of both P‐selectin and PAC1 were significantly reduced (*p *< 0.05) after treatment with DBMSCs as well as with the DPMSCs, whereas CVMSCs have no effect on collagen‐induced platelet activation. HUVECs decreased expression levels of both the P‐selectin and the PAC1 in collagen‐treated platelets. This phenotype was not observed in resting platelets, irrespective of their treatment with cells or untreated controls (Figure 3A and B).

Contrarily, pMSCs had minimal effect on the expression levels of both P‐selectin and on PAC1 in platelets induced by ADP (supplementary figure S3A and C). HUVECs have significant (*p *< 0.05) dose‐dependent effect pertaining to reduced platelet activation after their induction with ADP. However, there was no effect on P‐selectin or PAC1 expression in washed platelets with or without treatment with pMSCs or HUVECs (Figure S3B–D).

### 3.3 pMSC‐platelet co‐culture reduces agonist‐induced platelet functions

**FIGURE 4:**
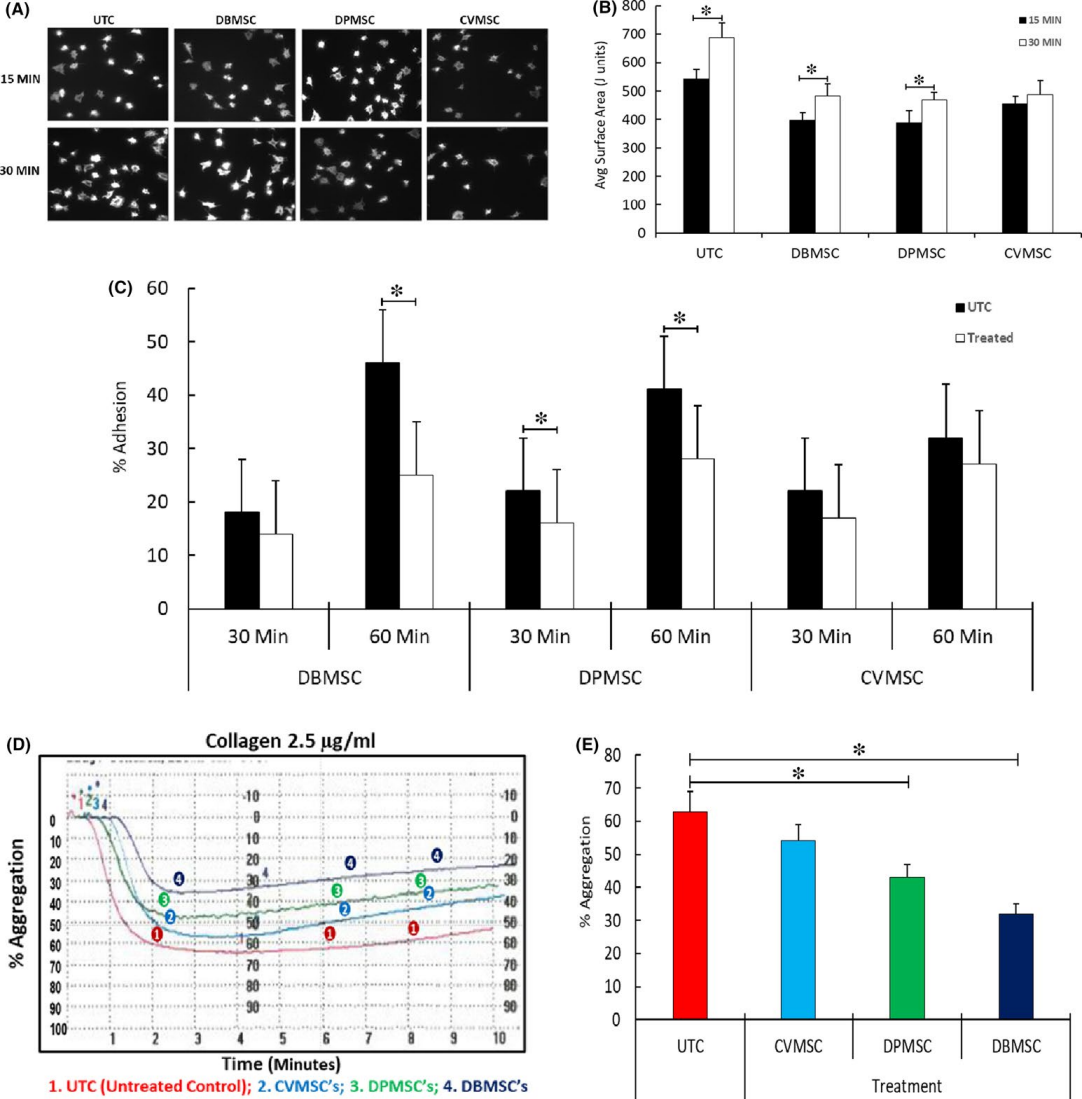
pMSC treatment reduces agonist dependent platelet functions: (A) washed platelets treated with DBMSCs, DPMSCs, CVMSCs or no treatment (untreated control) followed by collagen were added to fibrinogen‐coated coverslips for 15 and 30 min, and actin fibres were stained with Rhodamine Phalloidin. (B) The surface area of platelets spread on fibrinogen was quantified from 100 platelets. Data shown are an average of 3 experiments. (C) Static adhesion of pMSCs (DBMSCs, DPMSCs and CVMSCs)‐treated platelets for 15 and 30 min to 100 µg/ml of fibrinogen. (D) pMSCs‐treated platelets displayed reduced aggregation to low dose collagen. Washed platelets treated with pMSCs were challenged with collagen, and the light impedance was measured in an aggregometer. (E) Final percentage aggregation of platelets treated with different pMSCs and collagen is shown as a bar graph with ±SE from five independent experiments. pMSCs were isolated from 5 different placentae for each cell type used in the study. *p ≤ 0.05

As shown in Figure 4A, compared to the untreated controls, the DBMSCs, DPMSCs and the CVMSCs‐treated platelets showed an increase in filopodial extensions on immobilized fibrinogen, which was reflected by an increase in their surface area. These results were time‐dependent and were visualized at both 15 min and 30 min of treatment. Quantification of the surface area revealed that the difference between the untreated controls and the pMSCs‐treated platelets was statistically significant in at least three independently executed experiments (Figure 4B).

In a calorimetric adhesion assay, it was observed that platelet adhesion to immobilized fibrinogen was diminished after pMSCs treatment, when compared to untreated platelets under similar conditions (Figure 4B). Platelets treated with DBMSCs showed significant decreases in adhesiveness at 60 min of treatment, whereas platelets treated with DPMSCs showed significant decreases in adhesion at both time points viz., 30 min and 60 min. Although there was a decrease in platelet adhesion with CVMSCs‐treated platelets, yet the data were not statistically significant (Figure 4C).

As shown in Figure 4D, untreated controls and the platelets treated with CVMSCs had almost similar levels of aggregation to 2.5 µg of collagen. However, platelets treated with DPMSCs and DBMSCs with similar doses of collagen displayed decreased aggregation. Quantification of data on final aggregation showed that platelets treated with DPMSCs and DBMSCs exhibited significantly reduced aggregation as compared to the untreated controls (Figure 4E right panel).

Similar phenotypes were observed for other agonists, such as ADP and U46619, where treatment with DBMSCs and DPMSCs resulted in decreased platelet aggregation as compared to CVMSCs and untreated control (data not shown).

### 3.4 Differential expression of pro‐ and anti‐thrombotic factors in pMSCs

**FIGURE 5:**
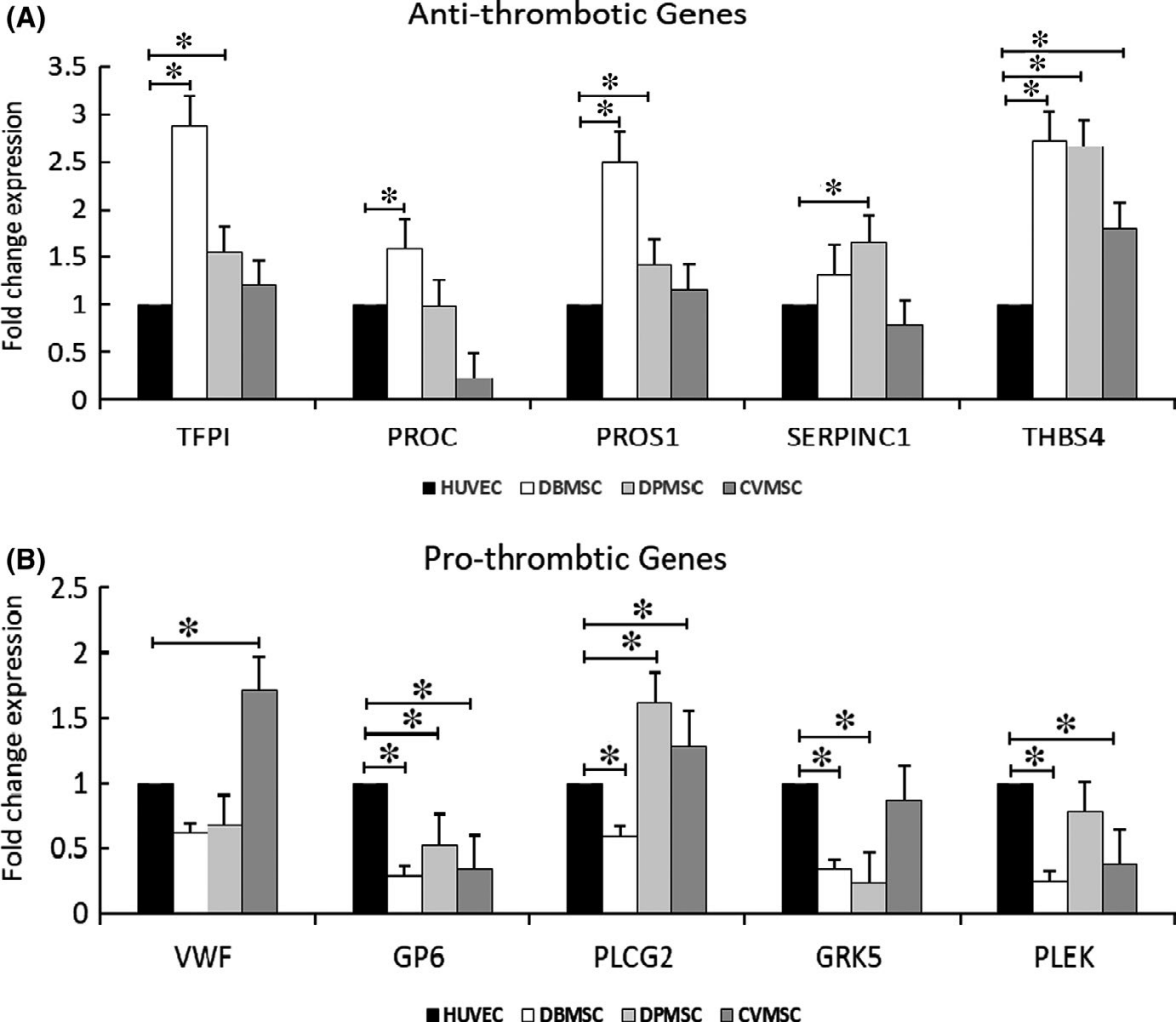
pMSCs demonstrate differential expression of genes involved in coagulation and thrombosis: Real‐time PCR analysis (RT‐PCR) analysis of pro‐ and anti‐thrombotic genes expressed in pMSCs in comparison to HUVECs. (A) Genes inhibiting thrombosis and coagulation. *TFPAI* (Tissue Factor Pathway Inhibitor), *PROC1* (Vitamin K‐dependent protein C1), *PROS1* (Protein S1), *SERPINC1* (Serpin Family C Member 1) and *THBS4* (Thrombospondin 4). (B) Genes promoting thrombosis and coagulation. *vWF* (Von Willebrand Factor), *GP6* (Glycoprotein VI platelet), *PLCG2* (Phospholipase C Gamma 2), *GRK5* (G Protein‐Coupled Receptor Kinase 5) and *PLEK* (Pleckstrin). Three independent experiments were performed for each cell type isolated from three different placentae. The data are expressed as fold change difference calculated from the ΔΔ^−2^ values. Bars represent mean ± SE. *p ≤ 0.05

As compared to the HUVECs, DBMSCs showed upregulated mRNA levels for anti‐thrombotic genes, such as *TFPAI*, *PROC1*, *PROS1*, *SERPINC1* and *THBS4* (Figure 5 and supplementary table S1). Similar pattern of expression of all genes was observed in DPMSCs except *PROC1*, the expression level of which was equal to the HUVECs. As compared to HUVECs, CVMSCs expressed lesser or equitable levels of *TFPI*, *PROS1* and *SERPINC1*. They show decreased expression of *PROC1* and significant increase in *THBS4* levels as compared to HUVECs (Figure 5A).

pMSCs expressed differential levels of pro‐thrombotic genes varying from cell types and as compared to the HUVECs (Figure 5B). DBMSCs expressed decreased levels of *vWF* and significant reduction in the levels of other pro‐thrombotic genes such as *GP6*, *PLCG2*, *GRK5* and *PLEK*. Similarly, as compared to HUVEC controls, DPMSCs expressed diminished levels of *vWF* and significant reduction in *GP6* and *GRK5* levels. However, significant increase in *PLCG2* and *PLEK* levels was observed in DPMSCs. In contrast, the *vWF* and the *PLCG2* levels were significantly higher in CVMSCs as compared to HUVECs controls. Although there was significant reduction in *GP6* and *PLEK* levels in CVMSCs, there was no traceable change in the expression levels of *GRK5* as compared to HUVECs (Figure 5B).

**FIGURE 6:**
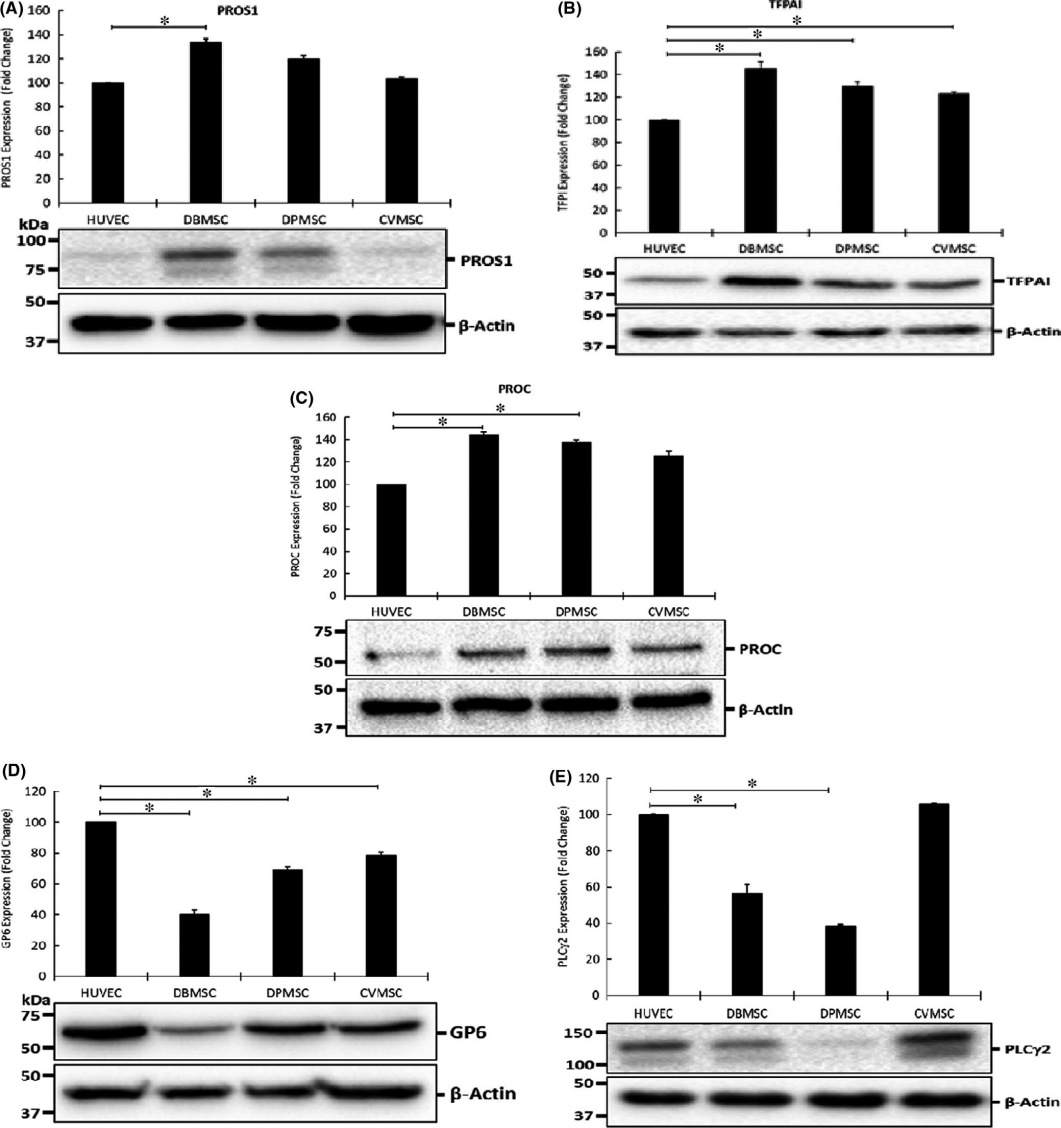
Validation of the RT‐PCR results by Immunoblotting: The RT‐PCR results obtained for the pro‐ and anti‐thrombotic genes were further verified at the protein level by immunoblotting. (A) PROS1; (B) TFPAI; (C) PROC1; (D) GP6 and (E) PLCG2. Bars ± SE represent the quantification of bands obtained by performing densitometry for each protein. *p ≤ 0.05

mRNA expression of pro‐ and anti‐thrombotic genes was further verified by their translation at the protein level. As shown in Figure 6A–C, and as compared to HUVECs, the differences in mRNA profiles of *PROS1*, *TFPAI*, *PROC1* and *GP6* showed differential expression levels for all the effectors analysed. However, PLCG2 was found to be downregulated exclusively in DPMSCs. Densitometry analysis of the bands further corroborated the data obtained by protein analysis (Figure 6A–C).

### 3.5 DBMSCs inhibit ox‐LDL‐induced‐CD36‐mediated platelet activation

**FIGURE 7:**
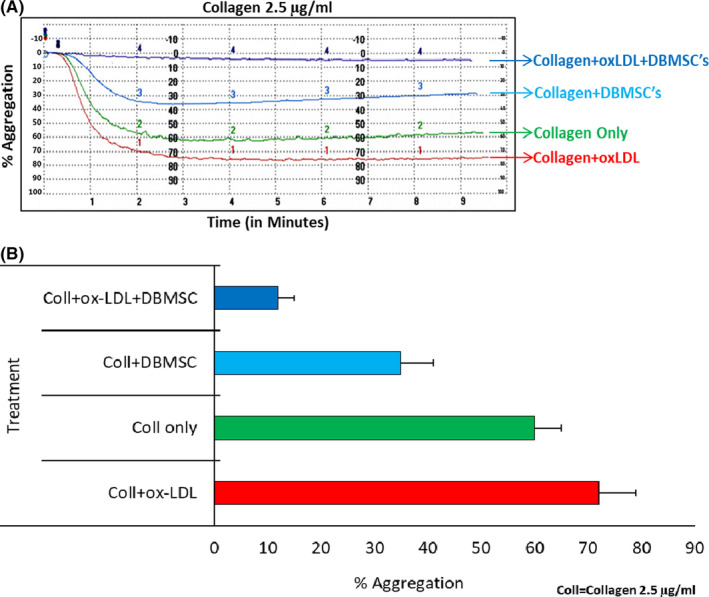
DBMSCs inhibit platelet activation induced by ox‐LDL and collagen: (A) Washed platelets were initially treated with DBMSCs for 10 min and subsequently incubated with 50 μM ox‐LDL (as per manufacturer's instructions) for 30 min. Collagen at 2.5 µg was added under stirring conditions, and aggregation was recorded. In comparison, other experimental conditions such as platelets treated with collagen and ox‐LDL, platelets treated with collagen only and platelets treated with DBMSCs and induced with collagen were included in the experiments. (B) Final percentage aggregation of platelets with different treatments is shown as a bar graph with ±SE from 3 independent experiments. *p ≤ 0.05

As shown in Figure 7A, collagen supplemented with ox‐LDL enhanced aggregation of washed platelets, as compared to collagen only treatment under stirring conditions. As observed in previous functional experiments, DBMSCs treatment reduced collagen‐induced aggregation of washed platelets. However, this decrease in platelet aggregation was further minimized in platelets incubated with DBMSCs and treated with collagen/ox‐LDL (Figure 7A). Figure 7B depicts the final percentage aggregation of platelets treated with ox‐LDL, collagen and with or without DBMSCs.

## 4 DISCUSSION

We have earlier reported the isolation and characterization of MSCs from decidua and villus regions of human term placenta and described them as *decidua basalis* (DB), *decidua parietals* (DP) and *chorionic villous* (CV) mesenchymal stem cells, collectively referred to as pMSCs.^28^, ^29^, ^30^ pMSCs deployed major histocompatibility complex (MHC) class I but not MHC class II, even after stimulation with interferon‐ɤ; (IFN‐ɤ), thus displaying immunomodulatory properties, which makes them useful therapeutic agents for immune‐mediated diseases.^31^ In order to utilize pMSCs as therapy against a particular disease, the transplantation/ injection of pMSCs is a routine process. In the circulation, they face multiple cells types, including T cells, NK cells, B cells, monocytes, DCs and the platelets. The immunomodulatory properties of MSCs have been extensively studied on various circulating cells including T cells, NK cells, B cells, monocytes, DCs and macrophages.^38^, ^39^, ^40^ Therapeutic potential of pMSCs to treat several inflammatory diseases, such as diabetes and cancer, was reported earlier.^28^, ^32^, ^38^, ^39^, ^40^, ^41^ Since atherosclerosis is also an inflammatory disease,^3^ we focussed on primary haemostasis and examined if pMSCs modulate platelet activation and help reduce plaque formation in atherosclerosis. Additionally, we sought to identify important molecules expressed by pMSCs, involved in modulating such platelet‐based haemostasis events.

With the aim of evaluating the functional changes in platelets after they were challenged with pMSCs or their secreted products, it was imperative to determine an appropriate dose of CM or an approximate number of cells, which has measurable outcome in a given time. Initially, platelets were treated with different concentrations of CM ranging from 5% to 20% followed by stimulation with various agonists, and platelet activation markers CD62P (platelet surface P‐selectin) and PAC1 (activated integrin α_IIb_β_3_) were measured. Treatment of platelets with different concentrations of CM from DBMSCs and DPMSCs significantly decreased expression of both P‐selectin and PAC1 in a dose‐dependent manner. This decrease in P‐selectin and PAC1 expression was not observed for CM of CVMSCs. Conditioned medium obtained from various types of pMSCs grown in culture (in vitro) contain plethora of factors including cytokines, chemokines, growth factors and necessary adhesion molecules, as described earlier.^28^, ^29^, ^30^ Expression of these molecules by DBMSCs and DPMSCs may block the receptors of the particular agonists responsible for physiological processes leading to platelet activation. Interestingly, the decrease in platelet activation was not observed for all the agonists used, rather in collagen and U46619‐treated platelets only. This outcome may be because of factors produced by pMSCs specifically block the collagen and thromboxane A2 receptors such as GPVI or α_2_β_1_ and TP receptors. Treatment with CM from DBMSCs, DPMSCs and CVMSCs did not show any effect on ADP‐stimulated platelets explaining that the secretome of pMSCs is lacking the factors that block ADP receptors, P_2_Y_1_, P2Y_12_ and P2X1. Our previous findings support these data where we have shown that pMSCs differ in their secretome, which vary between, DBMSCs, DPMSCs and CVMSCs. These factors are responsible for endothelial cell survival, apoptosis, injury, fibrosis formation and inflammation.^38^, ^39^

Next, we examined the relevance of our findings in a clinical setup, where MSCs are transplanted in patients for various diseases. In order to assess their impact on platelet activation, we investigated the effects of pMSCs on platelet physiology by performing cell‐to‐cell contact treatment assays followed by evaluation of platelet activation markers. Using various clinically relevant number of stem cells versus the platelets, different ratios were tested to examine the effect of pMSCs on platelet activation. At a ratio of 1:1, 1:2 and 1:5, DBMSCs and DPMSCs reduced, the agonist (collagen and thromboxane A2) induced activation of platelets in a dose‐dependent manner. However, significant reduction was observed at 1:2 and 1:5 ratios that corresponded to 2 × 10^6^ and 5 × 10^6^, pMSCs to the platelets. However, it has been shown that a minimum dose of 2.5 × 10^6^ CD34^+^ cells was necessary for successful engraftment for neutrophils and platelets, yet reinfusion of 5.0 × 10^6^ CD34^+^ cells resulted in prompt engraftment and was agreed to be the preferred target.^37^ However, as observed for the CM, the inhibitory effect of cell‐to‐cell contact was observed for DBMSC and DPMSC only and was limited to collagen and thromboxane A2 agonists only. CVMSCs as compared to HUVECs did not show any effect on collagen and thromboxane A2‐treated platelets. These data confirm the outcome observed for CM, and the surface expression may be somewhat similar to the secretome of pMSCs. We have previously reported that pMSCs show surface expression of several important molecules which could inhibit the agonist‐induced activation of platelets in a co‐culture setup.^38^, ^39^

In order to assess the effect of pMSCs on platelet hyperactivity post‐agonist treatment, we performed platelet functional analyses and confirmed the inhibitory role of pMSCs and their secreted products. Several parameters that describe platelet activation in diseased setup at the site of injury, or after agonist induction, include their interaction to other cells (leukocytes or endothelial cells), their adhesion to artificial or natural surfaces, the receptor conformational change, changes in their actin cytoskeleton, secretion of molecules from the granules and most importantly the platelet‐platelet aggregation.^42^ In classical platelet activation pathways, agonists, such as collagen, thrombin or ADP, interact with platelet surface receptors and initiate intracellular signalling events resulting in activation of integrins, which culminate at shape change, secretion of platelet granule contents and platelet aggregation.^43^ With regard to these functional outcomes after agonist and/or pMSCs treatment, we report that barring CVMSs, both DBMSCs and the DPMSCs, increased platelet spreading, decreased platelet adhesion and platelet aggregation significantly, as compared to the HUVEC controls. These data suggest that DBMSCs and DPMSCs, but not the CVMSCs, possess anti‐thrombotic activities. This differential outcome may be related to the particular niche from which each cell type was isolated. However, further investigation is required to decipher the mechanism of difference in the outcome of the pMSCs isolated from the same source, the placenta. As expected, these inhibitory phenotypes were not observed in platelet‐rich plasma (PRP) or washed platelets, treated with DBMSCs or DPMSCs and not treated with any agonist. These data negate the role of any platelet‐specific intrinsic factor responsible for inhibition of platelet activity, as observed in pMSCs‐treated and agonist‐induced platelets. However, a moderate increase in activation of resting platelets while treating them with CVMSCs may prove beneficial in other haemostasis setup and further suggest the specific role of each cell type studied. It further proves that CVMSCs therapy may not be beneficial in certain diseases associated with a risk of thromboembolism. These results are in agreement with the previously reported studies performed on platelets using CD133^+^ bone marrow, lipoaspirate and cord blood stem cells.^44^, ^45^

As previously reported, pMSCs display immunomodulatory properties making them useful therapeutic agents for various diseases.^31^ They show surface expression of important modulators and secrete an array of soluble factors that exert immune responses on T cells, B cells, monocytes and macrophages.^31^ Investigation on expression of various genes and their products associated with coagulation and anticoagulation revealed that DBMSCs and DPMSCs express significant levels of anti‐atherosclerosis and anti‐thrombotic genes.^46^ However, as compared to DBMSCs and DPMSCs these factors expressed differentially in CVMSCs, which justify their differential outcome in the current studies. However, more studies are needed to understand the role and mechanism of each molecule in inhibition of platelet activation induced by pMSCs. These results are in accordance with the previously published findings, where expression of key factors implicated in regulation of haemostasis was found to be differentially expressed in bone marrow and decidua MSCs.^47^

It has been demonstrated that ox‐LDL binds to platelets via CD36 and induces platelet activation.^21^ Since platelet hyperactivity is associated with atherosclerosis, thrombosis and other inflammatory conditions,^5^ we examined whether pMSCs treatment modulates the agonist/ox‐LDL‐induced platelet activation and helps reduce plaque formation in atherosclerosis. Our studies demonstrated that DBMSCs significantly reduced aggregation of platelets mediated by ox‐LDL and induced by collagen, as compared to collagen only induction (Figure 7). Recent studies have demonstrated that CD36 is expressed on platelets in addition to other mammalian cell types including the macrophages, DCs, adipocytes.^48^ It binds to ox‐LDL and activates a specific signalling cascade that induces platelet activation.^5^, ^49^ It has also been demonstrated that CD36 contributes to thrombus formation in response to vascular injury in mice confirming the role of this pathway in platelet activation and promoting atherosclerosis.^43^ Results obtained in our study provide strong support to the hypothesis that pMSCs treatment not only inhibits the agonist‐induced platelet activation but also significantly inhibits the lipid‐induced platelet aggregation, making them a potential candidate for atherosclerosis therapy.

## 5 CONCLUSION

Our studies primarily demonstrate that pMSCs provide a protective role in agonist‐induced platelet activation, inhibiting thrombosis and atherosclerosis. These preliminary results depict that pMSCs not only inhibit agonist‐induced platelet activation but DBMSCs also inhibit ox‐LDL‐induced‐CD36‐mediated platelet aggregation providing a direct link between pMSCs therapy, hyperlipidaemia and atherosclerosis. Taken together, our study provides strong evidence that pMSCs‐based therapy has a potential to be used against atherosclerosis. However, a comprehensive plan is imminent to understand the inhibitory mechanism and to confirm their therapeutic potential in animal models, before applying them in clinical trials.

## CONFLICT OF INTEREST

Authors declare that there is no conflict of interest.

## AUTHOR CONTRIBUTIONS

**Abdullah Al Subayyil:** Data curation (supporting); Investigation (supporting); Methodology (supporting); Resources (supporting); Validation (equal); Visualization (supporting). **Yasser S. Basmaeil:** Data curation (equal); Formal analysis (supporting); Investigation (supporting); Methodology (supporting); Resources (equal); Validation (equal); Writing‐original draft (supporting). **Reem Alenzi:** Investigation (supporting); Methodology (supporting); Resources (supporting); Validation (supporting). **Tanvir Khatlani:** Conceptualization (lead); Data curation (lead); Formal analysis (lead); Funding acquisition (lead); Investigation (lead); Methodology (lead); Project administration (lead); Resources (lead); Software (lead); Supervision (lead); Validation (lead); Visualization (lead); Writing‐original draft (lead); Writing‐review & editing (lead).

## ETHICAL APPROVAL AND CONSENT TO PARTICIPATE

The Institutional Review Board (IRB) at King Abdullah International Medical Research Centre (KAIMRC), Saudi Arabia, approved this study and the consent form. Placentae were obtained after delivery from uncomplicated human pregnancies at 38‐40 gestational weeks, after signing a consent form.

## CONSENT FOR PUBLICATION

All authors agree to publish this manuscript.

## AUTHOR DISCLOSURE STATEMENT

The authors declare no competing financial interests.

## Supporting information

Supplementary Material

## Data Availability

All data generated during this study are included in this manuscript.
