# Supplementary material for: Human Placental Mesenchymal Stem/Stromal cells (pMSCs) inhibit agonist‐induced platelet functions reducing atherosclerosis and thrombosis phenotypes
Source: J Cell Mol Med. 2021 Sep 18;25(19):9268–80. doi: 10.1111/jcmm.16848 (PMC8500971; doi:10.1111/jcmm.16848)
Supplement: Supplementary file 1 — Supplementary Material [file JCMM-25-9268-s001.docx]

**Human Placental Mesenchymal Stem/Stromal cells (pMSCs) inhibit agonist-induced platelet functions reducing atherosclerosis and thrombosis phenotypes**

Abdullah Al Subayyil^1^, Yasser S. Basmaeil^1^, Reem Alenzi^1^, and Tanvir Khatlani^1*^

**Supplementary Material and Methods**

**1. Isolation and culture of pMSCs**

MSCs were isolated from different regions of placenta as per the protocols established in our lab (1-4).

The *Decidua Basalis* MSCs (DBMSCs) were isolated from decidua region of the maternal side of human placenta (1, 4), with minor modifications. Briefly, 10 grams of the decidua tissue was dissected and finely minced. The minced suspension was digested using digestion solution containing 0.3% collagenase type II (cat# 17101-015, ThermoFischer Scientific, Saudi Arabia) in PBS, with DNAse and required antibiotics (Penicillin at 100µg/ml and Streptomycin at 100U/ml). After filtration, the suspension was treated with RBC Lysis Buffer (cat# 00-4333-57, ThermoFischer Scientific, Saudi Arabia) for 45 min at room temperature, and re-centrifuged at 300g for 5 min. The supernatant was discarded and the cells were washed and cultured in T25 flasks in complete DMEM-F12 culture medium. At 75% confluency, the cells were harvested, characterized and re-cultured. At 75% confluency, the cells were used in subsequent experiments.

*Decidua Parietalis* MSCs (DPMSCs) were isolated from the decidua parietalis region of the placenta, which forms a bridge between the main uterine cavity and the fetal membrane of the placenta. The cells were isolated according to the protocols published previously (2, 4). Briefly, 10 grams of choriodecidua tissue was separated from the amnion region and minced to make a suspension. The suspension was centrifuged at 300g for 5 min, the supernatant discarded, and the pellet was re-suspended in the RBC Lysis Buffer (cat # 00-4333-57, ThermoFischer Scientific, Saudi Arabia), and incubated at room temperature for 45 min. After centrifugation, the pellet was incubated in DMEM/F-12 medium containing 50% MSC grade FBS (specifically designed for culture of MSCs). Cells were harvested, characterized and cultured at 37 °C in a cell culture incubator. Cells at 75% confluency at passage 3 were used in subsequent experiments.

MSCs from *Chorionic Villi* region of the placenta (CVMSCs) were isolated using our previously published explant method (5). Briefly, about 40 mg in total wet weight of tissue was collected from the fetal chorionic villi of the placental tissue. It was washed with PBS and incubated overnight at 4 °C in DMEM/F-12 digestion solution containing 2.5% trypsin (Life Technologies), 270 unit/mL DNase (Life Technologies), and antibiotics (100U/L penicillin and 100μg/mL streptomycin). The tissue was washed with PBS, and cultured in complete DMEM/F-12 culture medium at 37 °C in a cell culture incubator. The migrated cells from the explants were harvested and characterized, before use in subsequent experiments.

**2. Isolation of HUVECs and collection of Conditioned Media (CM)**

Human Umbilical Vein Endothelial Cells (HUVEC) were isolated from umbilical cord veins using the methods published earlier (6). The umbilical veins were thoroughly washed with PBS followed by digestion with collagenase type II (cat# 17101-015, ThermoFischer Scientific, Saudi Arabia) containing PBS solution. They were incubated at 37 °C in a cell culture incubator for 25 min. The liberated HUVECs were collected and re-suspended in complete Endothelial Cell Growth Medium (cat# PCS-100-041™, ATCC, USA), and cultured at 37 °C in a cell culture incubator. The cells were characterized by flow cytometry before using in the subsequent studies.

For collection of conditioned medium (CM) from the MSCs, cells at passages 2-4 were seeded in a T75 flask and cultured in a cell culture incubator at 37 °C, as already described (7). At 75% confluency, the medium was removed, and the cells were washed with PBS and replenished with fresh 10% FBS containing DMEM/F-12 media. After 72 hours, the conditioned medium was collected, centrifuged and stored at -80 °C.

**3. Real-Time PCR (RT-PCR)**

Total RNA was isolated from pMSCs (DBMSCs; DPMSCs and CVMSCs) and the HUVECs using RNEasy mini kit (Qiagen, MD, USA). 4μg of the total RNA was transcribed into single-stranded cDNA, using the Fastlane cDNA Analysis Kit (Qiagen, MD, USA). RT-PCR was performed using SYBR Green in the reaction mixture, and the samples were run in triplicate on the CFX96 RT-PCR detection system (Bio-Rad, CA, USA). Primer sequences of the genes analyzed in this study are shown in supplementary table 1. Preliminary data analysis was performed using the CFX Manager Software (Bio-Rad, CA, USA) and further analysis was done in Microsoft Excel. Results are expressed as fold change calculated from the ΔΔ^-2^ values. Expression of internal housekeeping gene (β-Actin) was used as a loading control. Each experiment was performed from cells at passage 3, prepared from three individual placentae.

**4. Immunoblotting**

The cells were washed twice with PBS to remove dead cells and debris. 100μl of cell lysis buffer (Cell Signaling Technologies, Beverly, MA) containing protease and phosphatase inhibitors was added to the dish and the cells were incubated for 10 min, before being scraped using a cell scrapper. Cell lysate was collected and centrifuged at 15,000 rpm for 5 min at 4 °C, and the supernatant was collected and stored at -80 °C, until further use. Protein quantity was estimated by Bradford assay method before use. For SDS-PAGE, 30μg of extracted protein was mixed with an equal amount of 2X Laemmli sample buffer (Bio-Rad, Hercules, CA, USA), boiled for 10 min and loaded onto a 10% SDS Polyacrylamide Gel (SDS-PAGE). The resolved proteins were transferred onto a Polyvinylidene Difluoride (PVDF) membrane using Mini Trans blot system (Bio-Rad, Hercules, CA, USA). The membranes were blocked for 30 min at room temperature (RT) with Tris-buffered saline containing 0.1% (v/v) Tween 20 (TBS-T) and 5% non-fat dry milk (Bio-Rad, Hercules, CA, USA). After blocking, the membranes were incubated overnight with protein specific primary antibodies at 1:1000 dilutions at 4 °C. The membranes were washed three times with TBS-T and incubated with horseradish peroxidase (HRP)-conjugated species-specific secondary antibodies (R&D Systems, Minneapolis MN) at 1:3000 dilution for 2 hours at RT. After washing the membrane three times with TBS-T, the protein bands were visualized using SuperSignal™ West Pico or West Femto Chemiluminescent Substrate (ThermoFischer Scientific, Saudi Arabia) in a ChemiDoc visualization system (Bio-Rad, Hercules, CA, USA). Differences in the band density was measured using the image analyzing software Image Lab (Bio-Rad, Hercules, CA, USA), while normalizing the values obtained for the test samples with the values obtained for β-Actin. The experiments were repeated three times, using cells from passage 3 isolated from different placentae.

**5. Statistical Analysis**

The statistical analysis of the data obtained for platelet activation in flow cytometry, is presented as mean fluorescence intensity (MFI). Results are presented as mean ± SEM and at least three individual experiments were performed to exclude the experimental variation. For statistical significance of the data, Student’s t test was performed using Graph-Pad Prism 8 (GraphPad Software, La Jolla, USA), and a p value of ≤ 0.05 was considered to be statistically significant.

**References:**

1. Abomaray FM, Al Jumah MA, Alsaad KO, Jawdat D, Al Khaldi A, AlAskar AS, Al Harthy S, Al Subayyil AM, Khatlani T, Alawad AO, Alkushi A, Kalionis B, Abumaree MH. Phenotypic and Functional Characterization of Mesenchymal Stem/Multipotent Stromal Cells from Decidua Basalis of Human Term Placenta. Stem Cells Int. 2016; 2016:5184601. doi: 10.1155/2016/5184601. Epub 2016 Feb 10. PMID: 27087815; PMCID: PMC4764756.

2. Abumaree MH, Abomaray FM, Alshehri NA, Almutairi A, AlAskar AS, Kalionis B, Al Jumah MA. Phenotypic and Functional Characterization of Mesenchymal Stem/Multipotent Stromal Cells From Decidua Parietalis of Human Term Placenta. Reprod Sci. 2016 Sep;23(9):1193-207. doi: 10.1177/1933719116632924. Epub 2016 Feb 22. PMID: 26902429.

3. Abumaree MH, Al Jumah MA, Kalionis B, Jawdat D, Al Khaldi A, AlTalabani AA, Knawy BA. Phenotypic and functional characterization of mesenchymal stem cells from chorionic villi of human term placenta. Stem Cell Rev Rep. 2013 Feb;9(1):16-31. doi: 10.1007/s12015-012-9385-4. PMID: 22628114.

4. In 't Anker PS, Scherjon SA, Kleijburg-van der Keur C, de Groot-Swings GM, Claas FH, Fibbe WE, Kanhai HH. Isolation of mesenchymal stem cells of fetal or maternal origin from human placenta. Stem Cells. 2004;22(7):1338-45. doi: 10.1634/stemcells.2004-0058. PMID: 15579651.

5. Castrechini NM, Murthi P, Qin S, Kusuma GD, Wilton L, Abumaree M, Gronthos S, Zannettino A, Gude NM, Brennecke SP, Kalionis B. Decidua parietalis-derived mesenchymal stromal cells reside in a vascular niche within the choriodecidua. Reprod Sci. 2012 Dec;19(12):1302-14. doi: 10.1177/1933719112450334. Epub 2012 Aug 10. PMID: 22886285.

6. Abumaree MH, Al Jumah MA, Kalionis B, Jawdat D, Al Khaldi A, Abomaray FM, Fatani AS, Chamley LW, Knawy BA. Human placental mesenchymal stem cells (pMSCs) play a role as immune suppressive cells by shifting macrophage differentiation from inflammatory M1 to anti-inflammatory M2 macrophages. Stem Cell Rev Rep. 2013 Oct;9(5):620-41. doi: 10.1007/s12015-013-9455-2. PMID: 23812784.

7. Alshabibi MA, Khatlani T, Abomaray FM, AlAskar AS, Kalionis B, Messaoudi SA, Khanabdali R, Alawad AO, Abumaree MH. Human decidua basalis mesenchymal stem/stromal cells protect endothelial cell functions from oxidative stress induced by hydrogen peroxide and monocytes. Stem Cell Res Ther. 2018 Oct 25;9(1):275. doi: 10.1186/s13287-018-1021-z. PMID: 30359307; PMCID: PMC6202803.

**Supplementary figures**

**Figure legends:**

**Suppl. Table 1. Sequence of primers used for RT-PCR analysis**. Abbreviations: TFPAI (Tissue Factor Pathway Inhibitor), PROC1 (Vitamin K-dependent protein C), PROS1 (Protein S), SERPINC1 (Serpin Family C Member 1), THBS4 (Thrombospondin 4), vWF (Von Willebrand Factor), GP6 (Glycoprotein VI platelet), PLCG2 (Phospholipase C Gamma 2), GRK5 (G Protein-Coupled Receptor Kinase 5) and PLEK (Pleckstrin)

**Suppl. Fig. 1. Conditioned media of pMSCs inhibit platelet activation:** Washed platelets incubated with conditioned medium (CM) at 5%, 10% and 20% concentration and then induced with ADP. Flow cytometry was performed to assess the expression of activation markers, P-selectin (CD62P) and PAC1, respectively. A. CM-DBMSCs; B. CM-DPMSCs; C. CM-CVMSCs; D. CM-HUVECs. Data is expressed as Mean Fluorescent Index (MFI). Platelet MFI after treatment with different CM concentrations and agonists is compared with CM and ADP untreated and washed platelets (Rest) as negative and ADP treated platelets as positive control. Bars with standard error (± SE) are representative of at least three experiments with pMSCs isolated from at least five different placentae. *P≤0.05

**Suppl. Fig. 2. Dose response for pMSCs treatment on platelet activation:** Platelets were co-cultured (intracellular treatment IC) with different number of pMSCs corresponding to 1:1, 1:2 and 1:5 ratio between platelets and stem cells, and subsequently induced with ADP at 5µM or left as untreated controls. The activation of platelets was assessed by flow cytometry for activation marker P-selectin (CD62P). Dose effect of pMSCs treatment on platelet activation as compared to MSC untreated and ADP treated and resting platelets. A. DBMSCs; B. DPMSCs; C and D. CVMSCs and HUVECs respectively. Data is reflected as MFI. Data is represented with as bars ± SE. Figure is representative of three individual experiments. pMSCs isolated from 5 different placentae for each cell type were used in the study. *P≤0.05.

**Suppl. Fig.3. A subset of pMSCs inhibit platelet activation:** Platelets co-cultured with or without (w/o) pMSCs/ HUVECs followed by activation with ADP or the resting (untreated) followed by flow cytometry for activation markers P-selectin (CD62P) and PAC1. P-selectin (CD62P) expression in MFI for: A. pMSCs/ HUVECs treated and ADP induced, B. pMSCs/ HUVECs treated and resting platelets. PAC1 expression in MFI for: C. pMSCs/ HUVECs treated, ADP induced, D. pMSCs treated, and resting platelets. Data is represented as bars with SE. Figure is representative of three separate experiments performed with pMSCs or HUVECs isolated from 5 different placentae for each cell type used in the study. *P≤0.05. UTC (Untreated Cellular Control).

Suppl Table 1.


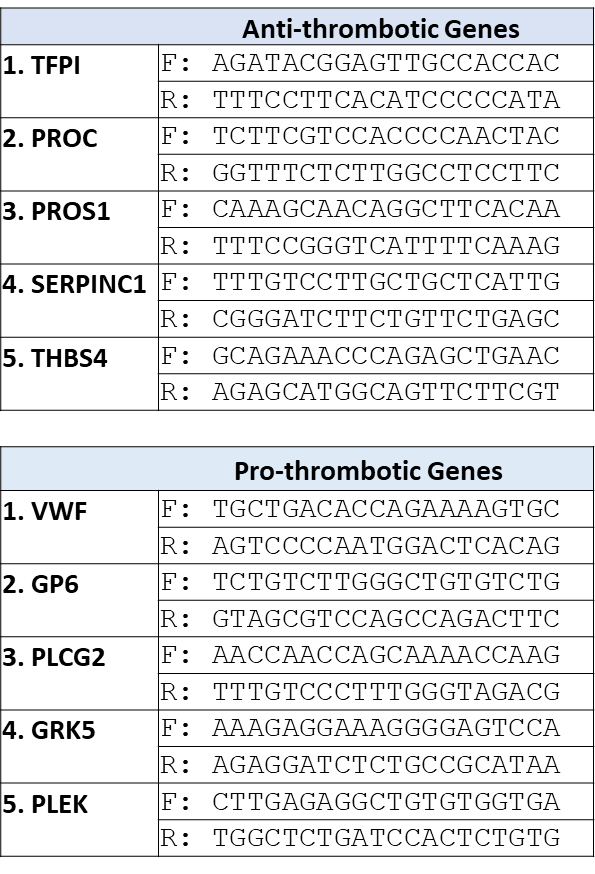


Suppl Fig. 1


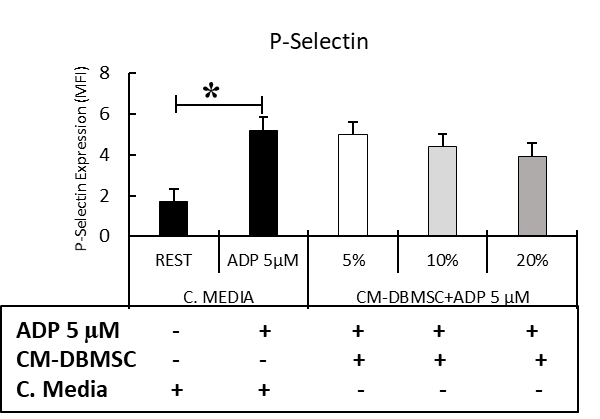

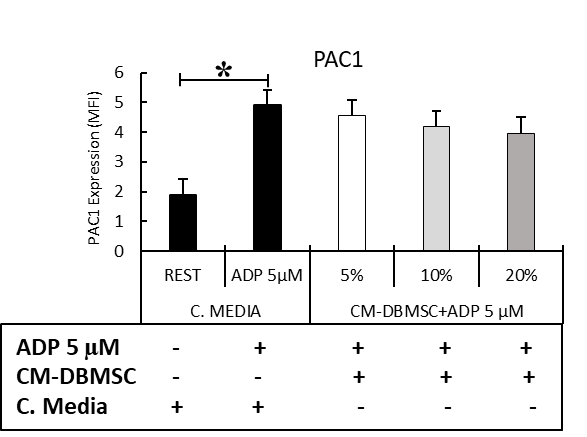

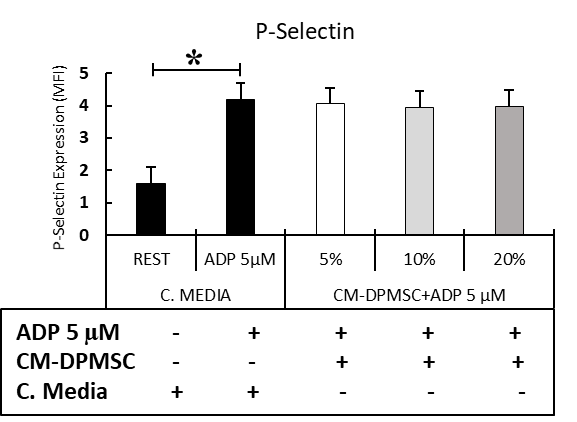

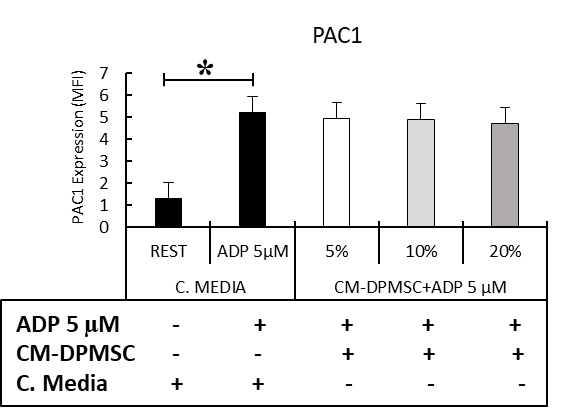

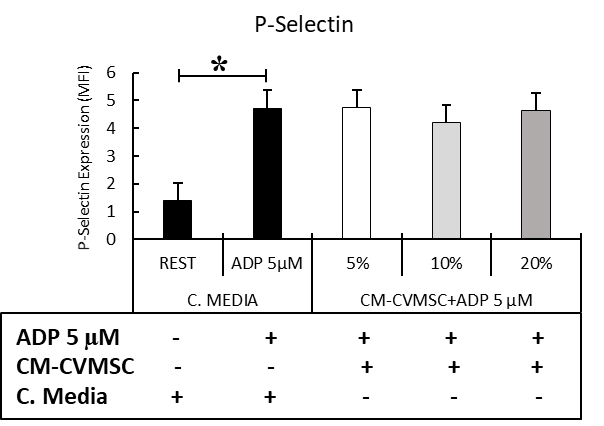

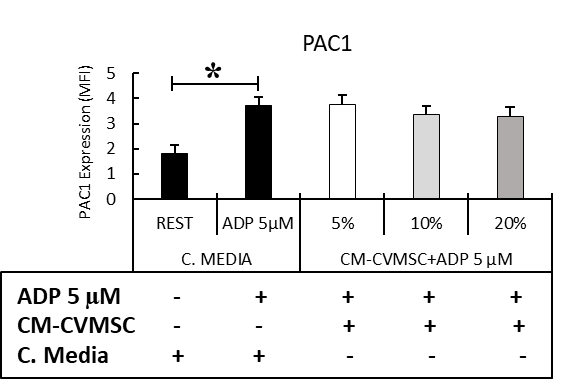

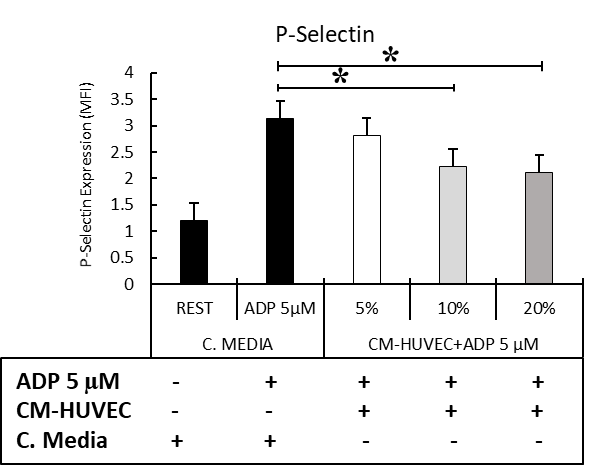

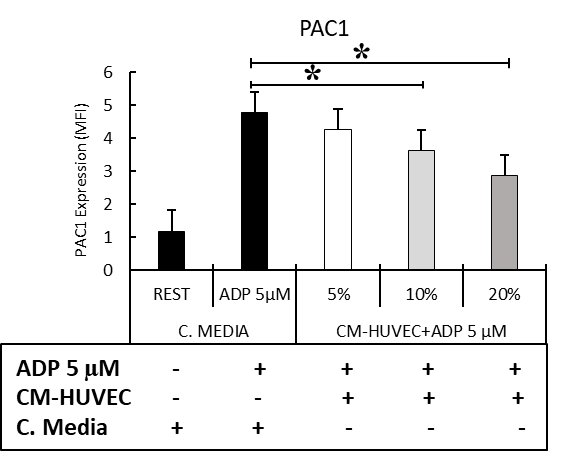


**A**

**B**

**C**

**D**

Suppl. Fig. 2


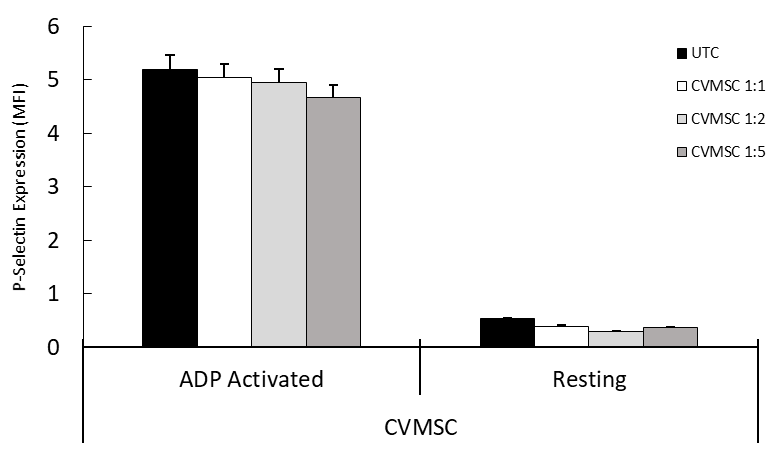

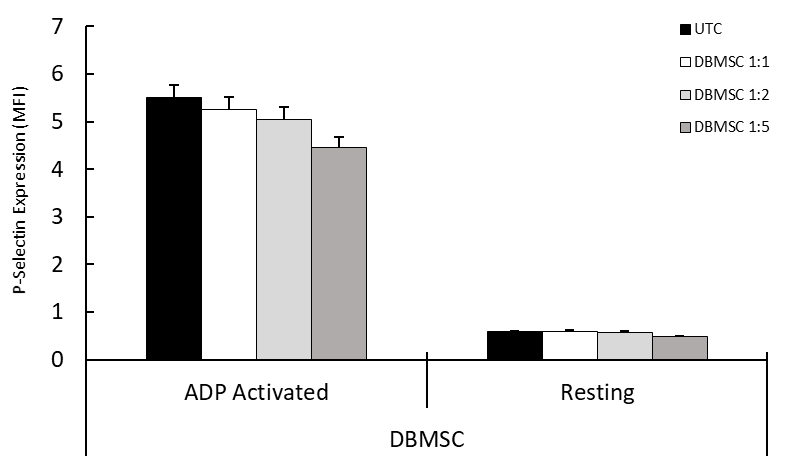

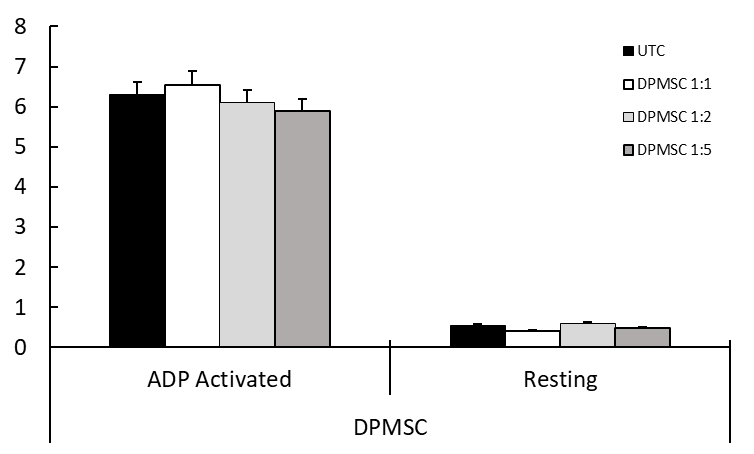

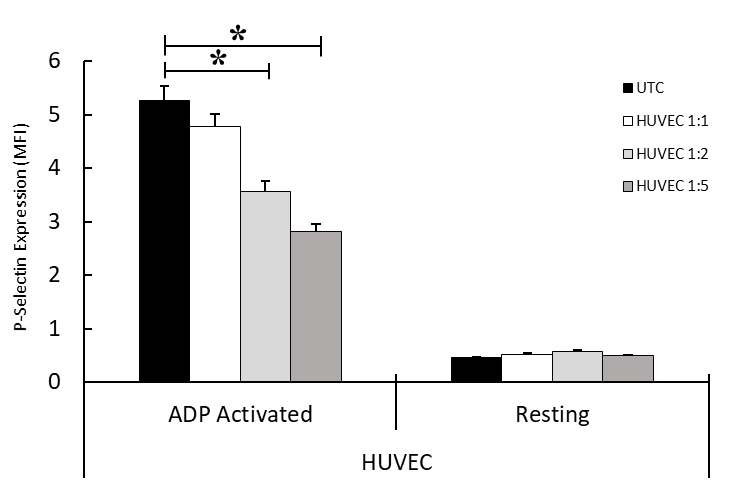


**A**

**B**

**C**

**D**

Suppl Fig 3.


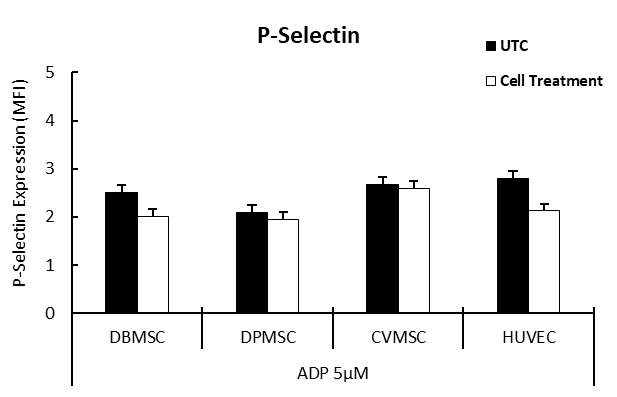

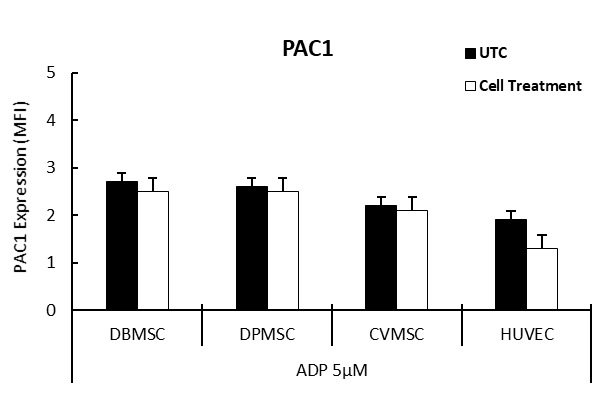

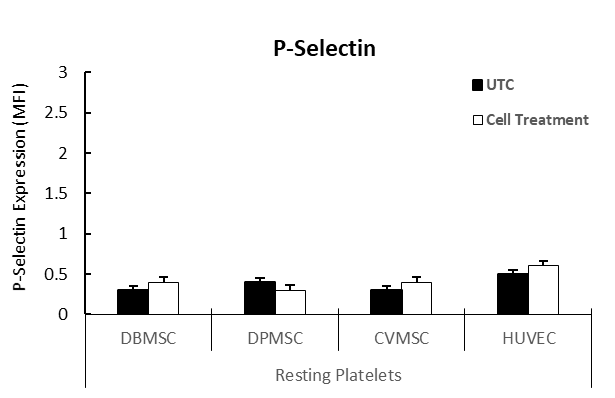

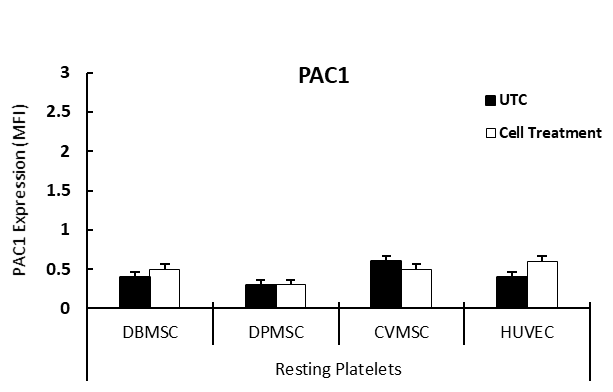


**A**

**B**

**C**

**D**
